# The genome sequence of a muscid fly, *Polietes domitor *(Harris, 1780)

**DOI:** 10.12688/wellcomeopenres.20648.1

**Published:** 2024-02-19

**Authors:** Steven Falk, Duncan Sivell, Judy Webb, Andrzej Grzywacz, University of Oxford and Wytham Woods Genome Acquisition Lab, Natural History Museum Genome Acquisition Lab, Darwin Tree of Life Barcoding collective, Wellcome Sanger Institute Tree of Life Management, Samples and Laboratory team, Wellcome Sanger Institute Scientific Operations: Sequence Operations, Wellcome Sanger Institute Tree of Life Core Informatics team, Tree of Life Core Informatics collective, Darwin Tree of Life Consortium

**Affiliations:** 1 Independent researcher, Kenilworth, England, UK; 2 Natural History Museum, London, England, UK; 3 Nicolaus Copernicus University in Toruń, Toruń, Poland

**Keywords:** Polietes domitor, muscid fly, genome sequence, chromosomal, Diptera

## Abstract

We present a genome assembly from an individual female *Polietes domitor* (muscid fly; Arthropoda; Insecta; Diptera; Muscidae). The genome sequence is 1,043.3 megabases in span. Most of the assembly is scaffolded into 6 chromosomal pseudomolecules, including the X sex chromosome. The mitochondrial genome has also been assembled and is 19.95 kilobases in length.

## Species taxonomy

Eukaryota; Metazoa; Eumetazoa; Bilateria; Protostomia; Ecdysozoa; Panarthropoda; Arthropoda; Mandibulata; Pancrustacea; Hexapoda; Insecta; Dicondylia; Pterygota; Neoptera; Endopterygota; Diptera; Brachycera; Muscomorpha; Eremoneura; Cyclorrhapha; Schizophora; Calyptratae; Muscoidea; Muscidae; Muscinae; Muscini; *Polietes*; *Polietes domitor* (Harris, 1780) (NCBI:txid2866287).

## Background

*Polietes domitor* is a muscid (Diptera: Muscidae) species currently classified under the genus *Polietes* Rondani, 1866 ( Pont, 1986). However, the systematic position of the species is not fully resolved, and alternative hypotheses consider the species should be placed in a genus *Pseudomorellia* Ringdahl, 1922 ( Nihei & Carvalho, 2007) or retained within the genus *Polietes*, possibly within a subgenus *Pseudomorellia* ( Shinonaga, 2003).

*Polietes domitor* has a Palaearctic distribution, where it is present in the Azores, from Portugal and the Archipelago of Britain and Ireland through Russia, China to Japan, northwards to northern Scotland, Lapland, and Yakutia ( Pont, 1986). In Britain, *P. domitor* is recognised as a common species, with a flight period mostly from May to September ( d’Assis Fonseca, 1968; NBN Atlas Partnership, 2023), yet in continental Europe it is not too common and becomes increasingly rare towards the Mediterranean region ( Gregor *et al.*, 2002). Adult insects are associated with animal carrion and faeces, while larvae develop exclusively in the dung of large herbivorous mammals. In the third instar, larvae may become highly predacious, preying on other larvae ( Skidmore, 1985), and *P. domitor* has been considered an important biological control agent of other concomitant muscid species. However, *P. domitor* larvae are not obligatory carnivores and may reach maturity even without access to a living prey.

The provided genome herein will serve as a source of information for phylogenomic purposes and may be used in comparative genomic studies to answer questions regarding biological adaptations in *Polietes* and the muscid subfamily Muscinae.

We present a chromosomally complete genome sequence for *Polietes domitor*, based on one female specimen from Wytham Woods, as part of the Darwin Tree of Life Project. This project is a collaborative effort to sequence all named eukaryotic species in the Atlantic Archipelago of Britain and Ireland.

## Genome sequence report

**Figure 1.**
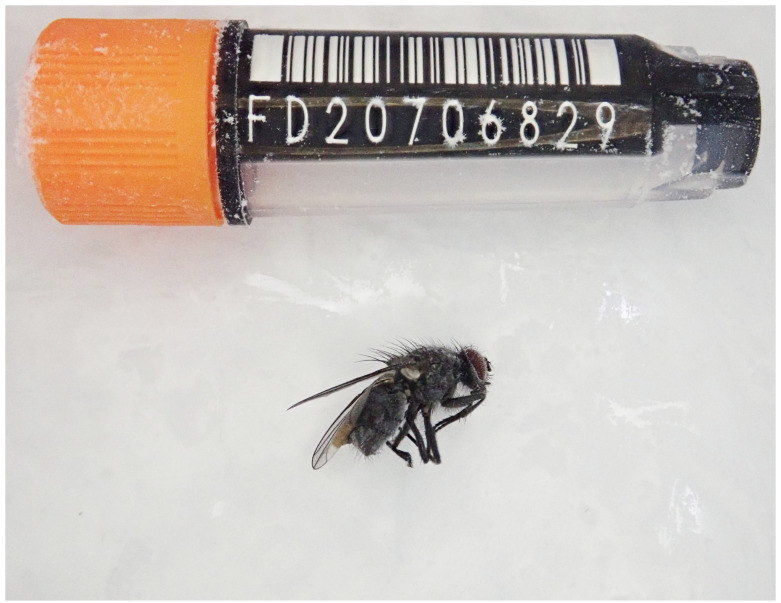
Photograph of the *Polietes domitor* (idPolDomi1) specimen used for genome sequencing.

The genome was sequenced from one female *Polietes domitor* ( Figure 1) collected from Wytham Woods, Oxfordshire, UK (51.76, –1.34). A total of 27-fold coverage in Pacific Biosciences single-molecule HiFi long reads was generated. Primary assembly contigs were scaffolded with chromosome conformation Hi-C data. Manual assembly curation corrected 154 missing joins or mis-joins and removed 22 haplotypic duplications, reducing the assembly length by 5.16% and the scaffold number by 20.83%, and increasing the scaffold N50 by 17.93%.

**Figure 2.**
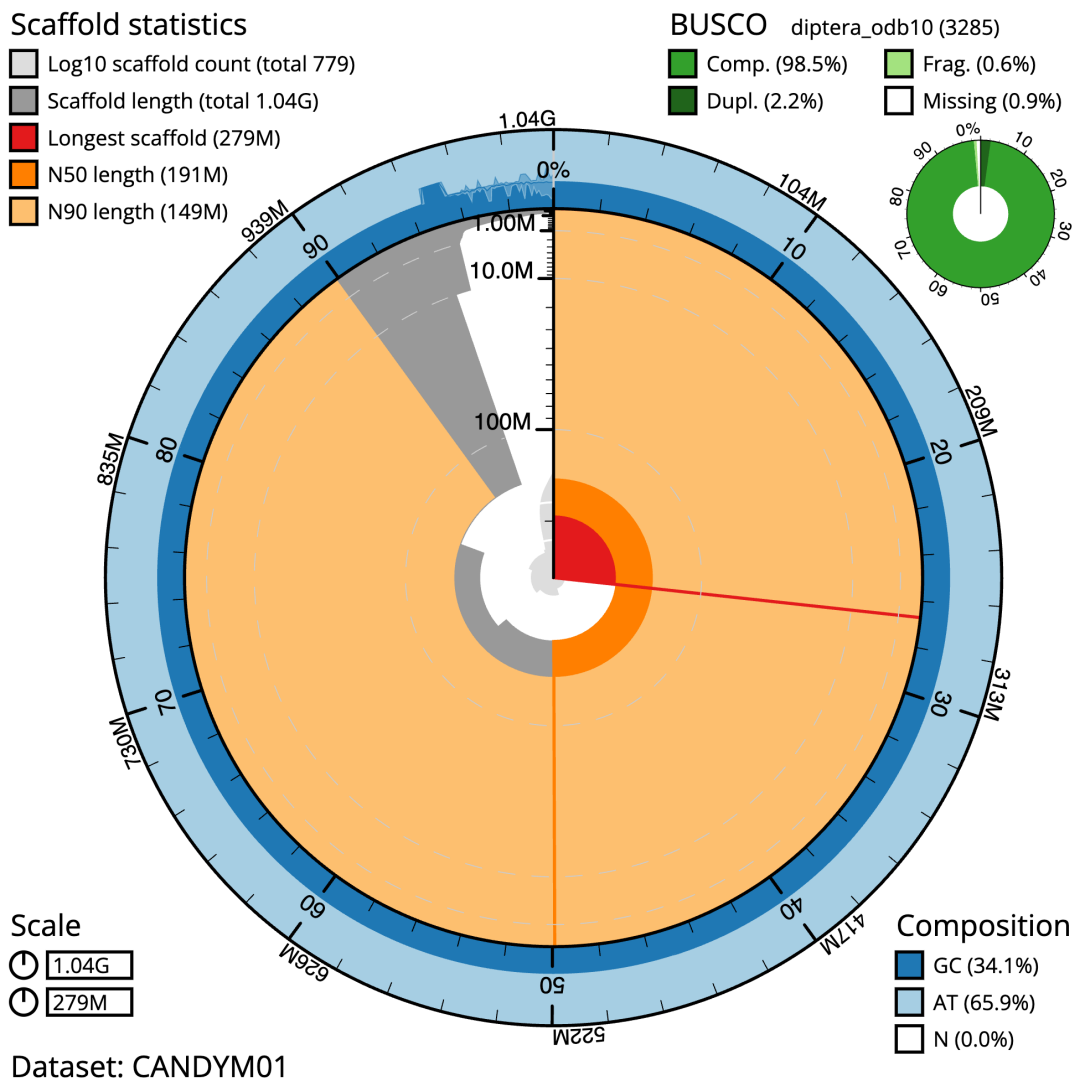
Genome assembly of *Polietes domitor*, idPolDomi1.1: metrics. The BlobToolKit Snailplot shows N50 metrics and BUSCO gene completeness. The main plot is divided into 1,000 size-ordered bins around the circumference with each bin representing 0.1% of the 1,043,329,465 bp assembly. The distribution of scaffold lengths is shown in dark grey with the plot radius scaled to the longest scaffold present in the assembly (278,847,389 bp, shown in red). Orange and pale-orange arcs show the N50 and N90 scaffold lengths (190,665,199 and 149,011,708 bp), respectively. The pale grey spiral shows the cumulative scaffold count on a log scale with white scale lines showing successive orders of magnitude. The blue and pale-blue area around the outside of the plot shows the distribution of GC, AT and N percentages in the same bins as the inner plot. A summary of complete, fragmented, duplicated and missing BUSCO genes in the diptera_odb10 set is shown in the top right. An interactive version of this figure is available at https://blobtoolkit.genomehubs.org/view/Polietes%20domitor/dataset/CANDYM01/snail.

**Figure 3.**
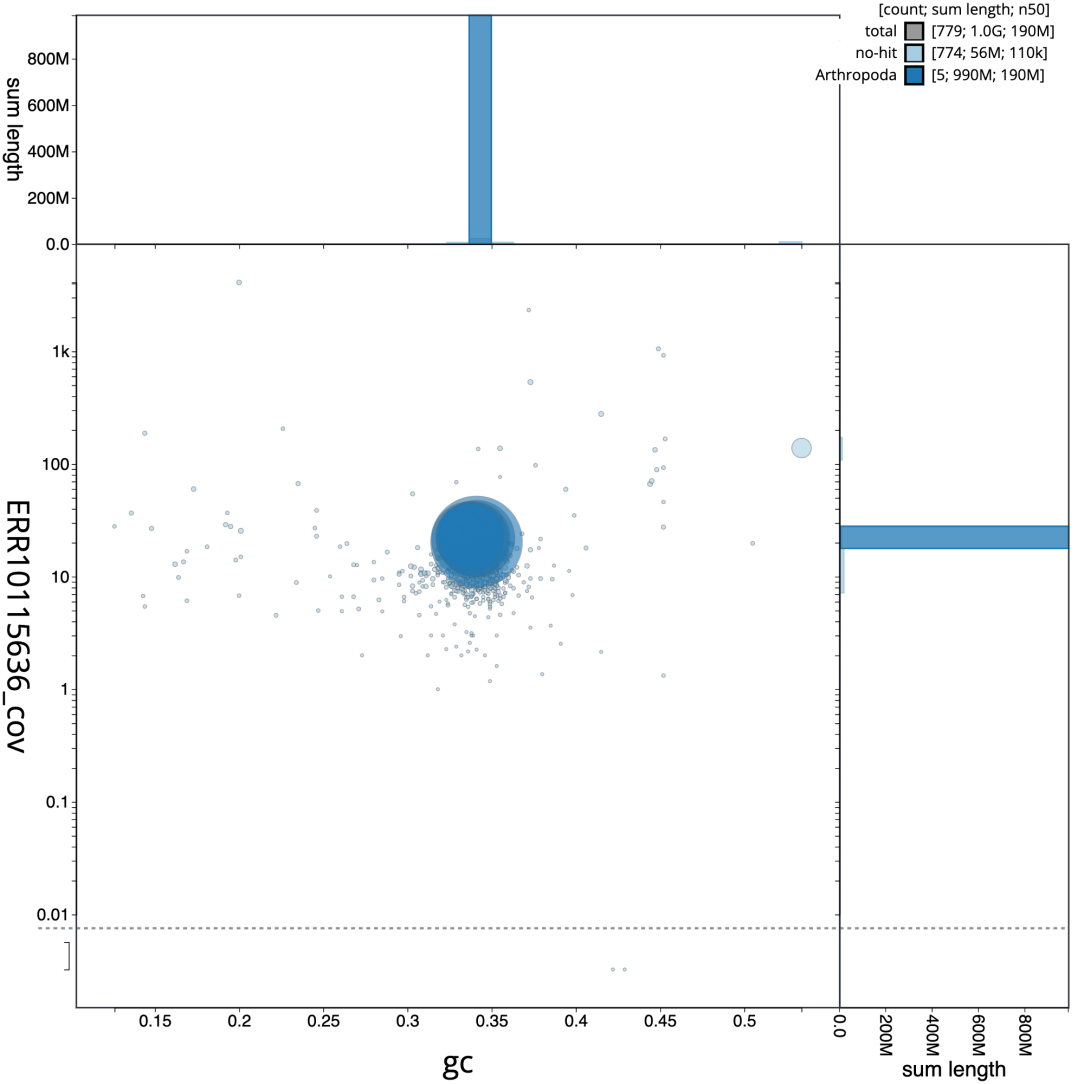
Genome assembly of *Polietes domitor*, idPolDomi1.1: BlobToolKit GC-coverage plot. Scaffolds are coloured by phylum. Circles are sized in proportion to scaffold length. Histograms show the distribution of scaffold length sum along each axis. An interactive version of this figure is available at https://blobtoolkit.genomehubs.org/view/Polietes%20domitor/dataset/CANDYM01/blob.

**Figure 4.**
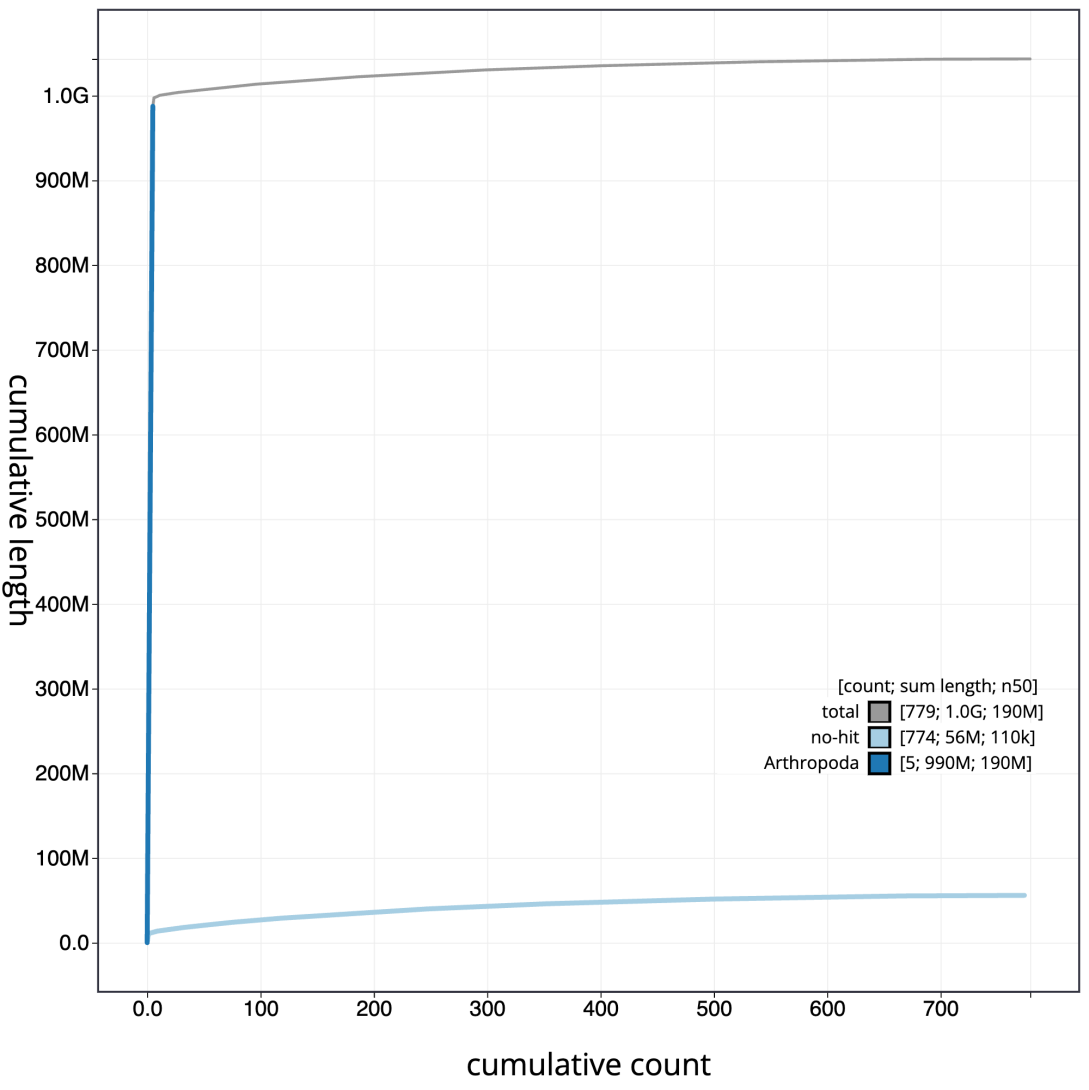
Genome assembly of *Polietes domitor*, idPolDomi1.1: BlobToolKit cumulative sequence plot. The grey line shows cumulative length for all scaffolds. Coloured lines show cumulative lengths of scaffolds assigned to each phylum using the buscogenes taxrule. An interactive version of this figure is available at https://blobtoolkit.genomehubs.org/view/Polietes%20domitor/dataset/CANDYM01/cumulative.

**Figure 5.**
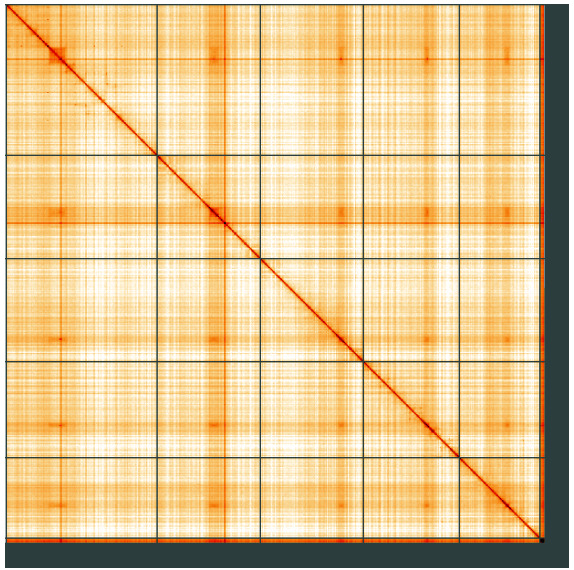
Genome assembly of *Polietes domitor*, idPolDomi1.1: Hi-C contact map of the idPolDomi1.1 assembly, visualised using HiGlass. Chromosomes are shown in order of size from left to right and top to bottom. An interactive version of this figure may be viewed at https://genome-note-higlass.tol.sanger.ac.uk/l/?d=Omg1UH2-QH26bpDmGk8uPA.

The final assembly has a total length of 1,043.3 Mb in 778 sequence scaffolds with a scaffold N50 of 190.7 Mb ( Table 1). The snailplot in Figure 2 provides a summary of the assembly statistics, while the distribution of assembly scaffolds on GC proportion and coverage is shown in Figure 3. The cumulative assembly plot in Figure 4 shows curves for subsets of scaffolds assigned to different phyla. Most (96.07%) of the assembly sequence was assigned to 6 chromosomal-level scaffolds, representing 5 autosomes and the X sex chromosome. Chromosome-scale scaffolds confirmed by the Hi-C data are named in order of size ( Figure 5; Table 2). While not fully phased, the assembly deposited is of one haplotype. Contigs corresponding to the second haplotype have also been deposited. The mitochondrial genome was also assembled and can be found as a contig within the multifasta file of the genome submission.

**Table 1.** Genome data for *Polietes domitor*, idPolDomi1.1.

Project accession data		
Assembly identifier	idPolDomi1.1	
Species	*Polietes domitor*	
Specimen	idPolDomi1	
NCBI taxonomy ID	2866287	
BioProject	PRJEB55492	
BioSample ID	SAMEA10166795	
Isolate information	idPolDomi1 (DNA sequencing) idPolDomi2 (Hi-C)	
Assembly metrics *		*Benchmark*
Consensus quality (QV)	58.9	*≥ 50*
*k*-mer completeness	100%	*≥ 95%*
BUSCO **	C:98.5%[S:96.3%,D:2.2%],F:0.6%, M:0.9%,n:3,285	*C ≥ 95%*
Percentage of assembly mapped to chromosomes	96.07%	*≥ 95%*
Sex chromosomes	X chromosome	*localised homologous pairs*
Organelles	Mitochondrial genome assembled	*complete single alleles*
Raw data accessions		
PacificBiosciences SEQUEL II	ERR10115636	
Hi-C Illumina	ERR10107967	
Genome assembly		
Assembly accession	GCA_947397865.1	
*Accession of alternate haplotype*	GCA_947397875.1	
Span (Mb)	1043.3	
Number of contigs	2499	
Contig N50 length (Mb)	1.2	
Number of scaffolds	778	
Scaffold N50 length (Mb)	190.7	
Longest scaffold (Mb)	278.9	

* Assembly metric benchmarks are adapted from column VGP-2020 of “Table 1: Proposed standards and metrics for defining genome assembly quality” from ( Rhie *et al.*, 2021). ** BUSCO scores based on the diptera_odb10 BUSCO set using v5.3.2. C = complete [S = single copy, D = duplicated], F = fragmented, M = missing, n = number of orthologues in comparison. A full set of BUSCO scores is available at https://blobtoolkit.genomehubs.org/view/Polietes%20domitor/dataset/CANDYM01/busco.

**Table 2.** Chromosomal pseudomolecules in the genome assembly of *Polietes domitor*, idPolDomi1.

INSDC accession	Chromosome	Length (Mb)	GC%
OX377618.1	1	278.85	34.0
OX377619.1	2	191.25	34.0
OX377620.1	3	190.67	33.5
OX377621.1	4	177.69	34.0
OX377622.1	5	149.01	33.5
OX377623.1	X	9.78	53.5
OX377624.1	MT	0.02	22.5

The estimated Quality Value (QV) of the final assembly is 58.9 with *k*-mer completeness of 100%, and the assembly has a BUSCO v5.3.2 completeness of 98.5% (single = 96.3%, duplicated = 2.2%), using the diptera_odb10 reference set ( *n* = 3,285).

Metadata for specimens, barcode results, spectra estimates, sequencing runs, contaminants and pre-curation assembly statistics are given at https://links.tol.sanger.ac.uk/species/2866287.

## Methods

### Sample acquisition and nucleic acid extraction

The specimen used for DNA sequencing was a female *Polietes domitor* (specimen ID Ox001318, ToLID idPolDomi1), netted in Wytham Woods, Oxfordshire (biological vice-county Berkshire), UK (latitude 51.76, longitude –1.34) on 2021-04-23. The specimen was collected and identified by Steven Falk (independent researcher) and snap-frozen on dry ice. The specimen used for Hi-C sequencing (specimen ID NHMUK014043031, ToLID idPolDomi2) was netted in Cothill Fen National Nature Reserve, England, UK (latitude 51.69, longitude –1.33) on 2021-04-25. The specimen was collected by Duncan Sivell and Judy Webb (Natural History Museum) and identified by Duncan Sivell and preserved on dry ice.

The workflow for high molecular weight (HMW) DNA extraction at the Wellcome Sanger Institute (WSI) includes a sequence of core procedures: sample preparation; sample homogenisation, DNA extraction, fragmentation, and clean-up, for which all protocols are available on protocols.io ( Denton *et al.*, 2023b).In sample preparation, the idPolDomi1 sample was weighed and dissected on dry ice ( Jay *et al.*, 2023). Tissue from the whole organism was homogenised using a PowerMasher II tissue disruptor ( Denton *et al.*, 2023a). HMW DNA was extracted in the WSI Scientific Operations core using the Automated MagAttract v2 protocol ( Oatley *et al.*, 2023). The extracted DNA was then sheared into an average fragment size of 12–20 kb in a Megaruptor 3 system with speed setting 31 ( Bates *et al.*, 2023). Sheared DNA was purified by solid-phase reversible immobilisation ( Strickland *et al.*, 2023): in brief, the method employs a 1.8X ratio of AMPure PB beads to sample to eliminate shorter fragments and concentrate the DNA. The concentration of the sheared and purified DNA was assessed using a Nanodrop spectrophotometer and Qubit Fluorometer and Qubit dsDNA High Sensitivity Assay kit. Fragment size distribution was evaluated by running the sample on the FemtoPulse system.

### Sequencing

Pacific Biosciences HiFi circular consensus DNA sequencing libraries were constructed according to the manufacturers’ instructions. DNA sequencing was performed by the Scientific Operations core at the WSI on a Pacific Biosciences SEQUEL II instrument. Hi-C data were also generated from head and thorax tissue of idPolDomi2 using the Arima2 kit and sequenced on the Illumina NovaSeq 6000 instrument.

### Genome assembly, curation and evaluation

Assembly was carried out with Hifiasm ( Cheng *et al.*, 2021) and haplotypic duplication was identified and removed with purge_dups ( Guan *et al.*, 2020). The assembly was then scaffolded with Hi-C data ( Rao *et al.*, 2014) using YaHS ( Zhou *et al.*, 2023). The assembly was checked for contamination and corrected as described previously ( Howe *et al.*, 2021). Manual curation was performed using HiGlass ( Kerpedjiev *et al.*, 2018) and Pretext ( Harry, 2022). The mitochondrial genome was assembled using MitoHiFi ( Uliano-Silva *et al.*, 2023), which runs MitoFinder ( Allio *et al.*, 2020) or MITOS ( Bernt *et al.*, 2013) and uses these annotations to select the final mitochondrial contig and to ensure the general quality of the sequence.

A Hi-C map for the final assembly was produced using bwa-mem2 ( Vasimuddin *et al.*, 2019) in the Cooler file format ( Abdennur & Mirny, 2020). To assess the assembly metrics, the *k*-mer completeness and QV consensus quality values were calculated in Merqury ( Rhie *et al.*, 2020). This work was done using Nextflow ( Di Tommaso *et al.*, 2017) DSL2 pipelines “sanger-tol/readmapping” ( Surana *et al.*, 2023a) and “sanger-tol/genomenote” ( Surana *et al.*, 2023b). The genome was analysed within the BlobToolKit environment ( Challis *et al.*, 2020) and BUSCO scores ( Manni *et al.*, 2021; Simão *et al.*, 2015) were calculated.

Table 3 contains a list of relevant software tool versions and sources.

**Table 3.** Software tools: versions and sources.

Software tool	Version	Source
BlobToolKit	4.1.7	https://github.com/blobtoolkit/blobtoolkit
BUSCO	5.3.2	https://gitlab.com/ezlab/busco
Hifiasm	0.16.1-r375	https://github.com/chhylp123/hifiasm
HiGlass	1.11.6	https://github.com/higlass/higlass
Merqury	MerquryFK	https://github.com/thegenemyers/MERQURY.FK
MitoHiFi	2	https://github.com/marcelauliano/MitoHiFi
PretextView	0.2	https://github.com/wtsi-hpag/PretextView
purge_dups	1.2.3	https://github.com/dfguan/purge_dups
sanger-tol/genomenote	v1.0	https://github.com/sanger-tol/genomenote
sanger-tol/readmapping	1.1.0	https://github.com/sanger-tol/readmapping/tree/1.1.0
YaHS	yahs-1.1.91eebc2	https://github.com/c-zhou/yahs

### Wellcome Sanger Institute – Legal and Governance

The materials that have contributed to this genome note have been supplied by a Darwin Tree of Life Partner. The submission of materials by a Darwin Tree of Life Partner is subject to the **‘Darwin Tree of Life Project Sampling Code of Practice’**, which can be found in full on the Darwin Tree of Life website here. By agreeing with and signing up to the Sampling Code of Practice, the Darwin Tree of Life Partner agrees they will meet the legal and ethical requirements and standards set out within this document in respect of all samples acquired for, and supplied to, the Darwin Tree of Life Project.

Further, the Wellcome Sanger Institute employs a process whereby due diligence is carried out proportionate to the nature of the materials themselves, and the circumstances under which they have been/are to be collected and provided for use. The purpose of this is to address and mitigate any potential legal and/or ethical implications of receipt and use of the materials as part of the research project, and to ensure that in doing so we align with best practice wherever possible. The overarching areas of consideration are:

•   Ethical review of provenance and sourcing of the material

•   Legality of collection, transfer and use (national and international)

Each transfer of samples is further undertaken according to a Research Collaboration Agreement or Material Transfer Agreement entered into by the Darwin Tree of Life Partner, Genome Research Limited (operating as the Wellcome Sanger Institute), and in some circumstances other Darwin Tree of Life collaborators.

## Data Availability

European Nucleotide Archive: *Polietes domitor*. Accession number PRJEB55492; https://identifiers.org/ena.embl/PRJEB55492 ( Wellcome Sanger Institute, 2022). The genome sequence is released openly for reuse. The *Polietes domitor* genome sequencing initiative is part of the Darwin Tree of Life (DToL) project. All raw sequence data and the assembly have been deposited in INSDC databases. The genome will be annotated using available RNA-Seq data and presented through the Ensembl pipeline at the European Bioinformatics Institute. Raw data and assembly accession identifiers are reported in Table 1.

## Author information

Members of the University of Oxford and Wytham Woods Genome Acquisition Lab are listed here: https://doi.org/10.5281/zenodo.7125292.

Members of the Natural History Museum Genome Acquisition Lab are listed here: https://doi.org/10.5281/zenodo.7139035.

Members of the Darwin Tree of Life Barcoding collective are listed here: https://doi.org/10.5281/zenodo.4893703.

Members of the Wellcome Sanger Institute Tree of Life Management, Samples and Laboratory team are listed here: https://doi.org/10.5281/zenodo.10066175.

Members of Wellcome Sanger Institute Scientific Operations: Sequencing Operations are listed here: https://doi.org/10.5281/zenodo.10043364.

Members of the Wellcome Sanger Institute Tree of Life Core Informatics team are listed here: https://doi.org/10.5281/zenodo.10066637.

Members of the Tree of Life Core Informatics collective are listed here: https://doi.org/10.5281/zenodo.5013541.

Members of the Darwin Tree of Life Consortium are listed here: https://doi.org/10.5281/zenodo.4783558.
