# Supplementary material for: The genome sequence of a muscid fly, Polietes domitor (Harris, 1780)
Source: Wellcome Open Res. 2024 Feb 19;9:58. [Version 1] doi: 10.12688/wellcomeopenres.20648.1 (PMC11411267; doi:10.12688/wellcomeopenres.20648.1)
Supplement: Peer review history

# Reviewer report: Reviewer response for version 1

**DOI:** 10.21956/wellcomeopenres.22852.r95132

**Published:** 2024-09-18

**Authors:** Ikechukwu Eugene Onah

**Affiliations:** 1 Department of Zoology and Environmental Biology, University of Nigeria, Nsukka, Enugu, Nigeria

The article provided  blue print of *Polietes domitor* genome. The study is important and will contribute in understanding the genomics and comparative genomics of the related flies. The availability of the genome will also enable understanding of the local adaptions of the species across different climatic regions.

 If this is the first genome of *P. domitor*, I suggest that the authors state it.

 The generic name is still in dispute, if this genome resolved the dispute you might state it in the article otherwise leave it as it is; the resolution could be published in a different manuscript when the genomes of closely related species becomes available for comparison.

Are sufficient details of methods and materials provided to allow replication by others?

Yes

Is the rationale for creating the dataset(s) clearly described?

Yes

Are the datasets clearly presented in a useable and accessible format?

Yes

Are the protocols appropriate and is the work technically sound?

Yes

Reviewer Expertise:

DNA barcoding, genomics

I confirm that I have read this submission and believe that I have an appropriate level of expertise to confirm that it is of an acceptable scientific standard.

# Reviewer report: Reviewer response for version 1

**DOI:** 10.21956/wellcomeopenres.22852.r77523

**Published:** 2024-04-11

**Authors:** Richard P Meisel

**Affiliations:** 1 University of Houston, Houston, Texas, USA

The authors generated a chromosome scale assembly of the female *Polietes domitor* genome, consisting of six chromosomes. Five of the chromosomes are inferred to be autosomal and one was assigned as the X chromosome, consistent with what would be expected for a calyptrate genome. My only suggestion for a worthwhile addition would be to assign each of the chromosomes to a "Muller element" Brachyceran genomes are organized into six chromosomal units (elements A-F), with element F representing the ancestral X chromosome. The authors have already assigned one chromosome to X=F. They could perform a homology search against Drosophila to assign the remaining chromosomes to their corresponding elements (A-E). This additional analysis would increase the value of this resource.

Are sufficient details of methods and materials provided to allow replication by others?

Yes

Is the rationale for creating the dataset(s) clearly described?

Yes

Are the datasets clearly presented in a useable and accessible format?

Yes

Are the protocols appropriate and is the work technically sound?

Yes

Reviewer Expertise:

Evolutionary genetics and genomics

I confirm that I have read this submission and believe that I have an appropriate level of expertise to confirm that it is of an acceptable scientific standard.
